# Case Report: Malignant transformation of ganglioneuroma in the rectovaginal septum to malignant peripheral nerve sheath tumor based on neurofibromas

**DOI:** 10.3389/fonc.2025.1566385

**Published:** 2025-06-19

**Authors:** Xiaoxiao Xi, Hui Cheng, Shanyu Huang, Chen Wang, Mingzhu Ye, Xin Sun

**Affiliations:** Department of Obstetrics and Gynecology, Third Xiangya Hospital, Central South University, Changsha, China

**Keywords:** malignant peripheral nerve sheath tumor, neurofibromatosis type 1, ganglioneuroma, rectovaginal septum, disorder of sex development, transvaginal surgery

## Abstract

Malignant peripheral nerve sheath tumor (MPNST) is a type of soft tissue sarcoma that commonly occurs in the trunk, limbs, and head and neck regions, but rarely in the pelvic area. Nearly half of MPNST cases are secondary to neurofibromatosis type 1 (NF1). This case report discusses a 29-year-old woman diagnosed with NF1 and pseudohermaphroditism, who presented with a large mass in the rectovaginal septum. Several years prior, a biopsy had identified the mass as a ganglioneuroma (GN). The mass was surgically removed via a vaginal approach, and postoperative pathology confirmed MPNST arising from NF1. In addition, this report provides a brief review of the relevant literature.

## 1 Introduction

Malignant peripheral nerve sheath tumor (MPNST) is an aggressive and rare sarcoma, accounting for 5%–10% of soft tissue sarcomas, with an incidence of approximately 0.001% (1, 2). Neurofibromatosis type 1 (NF1) is an autosomal dominant genetic disorder that is generally benign, with 8%–13% of cases potentially progressing to MPNST. It is characterized by multiple café-au-lait spots, freckling in the inguinal and axillary regions, Lisch nodules, and multiple neurofibromas. Clinical features, family history, and genetic testing are helpful for diagnosis (3). The prognosis of MPNST secondary to NF1 is poor. Treatment is primarily surgical, and negative surgical margins can significantly improve prognosis, with adjuvant therapies such as chemotherapy, radiotherapy, and immunotherapy (1). Ganglioneuroma (GN) is a mature form of neuroblastoma and a rare benign peripheral nerve tumor unrelated to genetics, with an incidence of 0.0001% (4). It commonly occurs in the sympathetic ganglia adjacent to the spine, with the posterior mediastinum being the most frequent site, followed by the retroperitoneum and pelvis (5). GN is more common in children, generally large in size, averaging 8 cm in diameter. It is often asymptomatic or causes compressive symptoms, although a small number may secrete active substances such as catecholamines, testosterone, or vasoactive intestinal peptides (6). Fine-needle aspiration aids the diagnosis of GN (7). Treatment may involve follow-up observation or surgical resection. The likelihood of malignant transformation is low, although a potential for transformation into neuroblastoma or MPNST exists (5). Some studies have suggested that diffuse GN may be associated with NF1 or NF2 (8).

## 2 Case description

### 2.1 Patient information

The patient, a 29-year-old woman, presented with a pelvic mass for 20 years and irregular menstruation for 1 year. At the age of 9, a pelvic mass measuring over 7 cm was identified, and she underwent exploratory laparotomy at a provincial hospital. During the surgery, the mass was found to be located deep in the retroperitoneal pelvic cavity, making excision difficult. A biopsy reported the mass as a GN, and no further pelvic mass excision was performed. Regular follow-ups showed gradual enlargement of the mass. At the age of 14, she began experiencing intermittent low back pain and left-sided sciatica, which were relieved with celecoxib. She visited several hospitals and all advised her to continue with regular follow-up because of the manageable symptoms and high surgical risks. In 2019, the patient presented with café-au-lait spots on the abdomen and back, and genetic testing confirmed NF1. In January 2023, she began to experience menstrual cycle irregularities, significantly increased menstrual flow, and eventually moderate to severe anemia. A hysteroscopy performed at a provincial hospital was unsuccessful due to the large vaginal mass, which prevented access to the uterine cavity. After a multidisciplinary team (MDT) discussion, she was started on “Uluet” treatment. However, during the course of treatment, she continued to experience intermittent heavy bleeding, followed by dizziness, fatigue, palpitations, shortness of breath, and frequent urination. Her past medical history showed that she was diagnosed with pseudohermaphroditism over 20 years ago without specific treatment, and had G1P1, with a cesarean section in 2019.

### 2.2 Specialist examinations

The vulva appeared asymmetrical bilaterally, with visible patches of brown pigmentation. The clitoris was enlarged, measuring approximately 4×3 cm. The left side of the vulva was notably more swollen than the right, extending toward the buttock. A large amount of yellow purulent discharge was observed in the vagina. The posterior vaginal wall was significantly raised, and a firm mass could be palpated, making it impossible to expose the upper part of the vagina or the cervix. On bimanual examination, a 10 cm firm mass was palpated between the posterior vaginal wall and the rectum, with an unclear upper border, and the cervix and uterus could not be palpated.

### 2.3 Auxiliary examinations after admission

#### 2.3.1 Routine inspections

Hemoglobin, 75 g/L; Testosterone, 88.81 ng/dL, with other sex hormones within normal range. Tumor markers: HE4, 81.4 pmol/L; CA724, 10.57 U/mL.

#### 2.3.2 Vaginal discharge culture

Escherichia coli was cultured, and it was sensitive to cefoperazone/sulbactam and other antibiotics.

#### 2.3.3 Transvaginal ultrasound:

**Figure 1:**
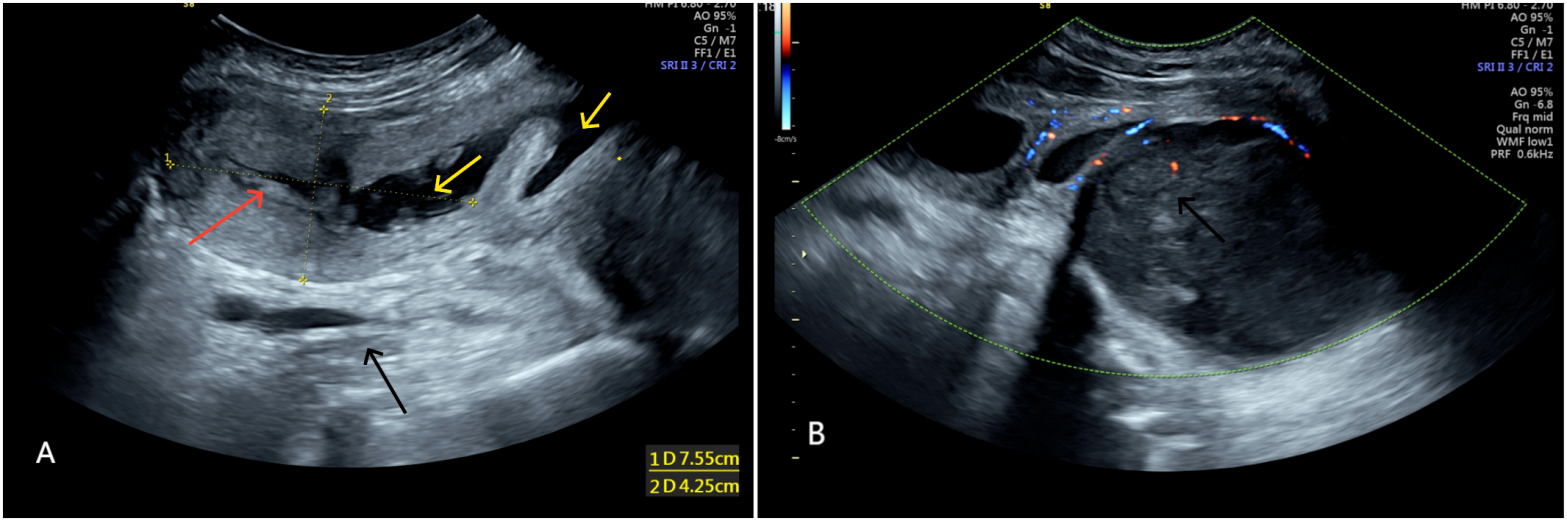
**(A)** Shows uterus (red arrow) displaced due to compression, fluid accumulation in uterine cavity and vagina (yellow arrow). **(A, B)** Show heterogeneous low echo mass (black arrow) ~116×85×112 mm behind the uterus.

- The uterus was measured approximately 75×42×62 mm. The endometrium in the upper segment of the uterine cavity was 6 mm thick, with uneven echogenicity.
- Fluid accumulation was noted: approximately 50×15×50 mm in the uterine cavity and cervical canal, and approximately 10 mm in width in the mid-upper vagina.
- Below and behind the uterus, a heterogeneous, low-echo mass, approximately 116×85×112 mm in size, was seen, which was closely related to the posterior vaginal wall. A fluid-filled area measuring approximately 50×27 mm was visible within the mass. The differential diagnosis included a fibroid of the vaginal wall or of retroperitoneal origin (**Figure 1**).

#### 2.3.4 Pelvic MRI

**Figure 2:**
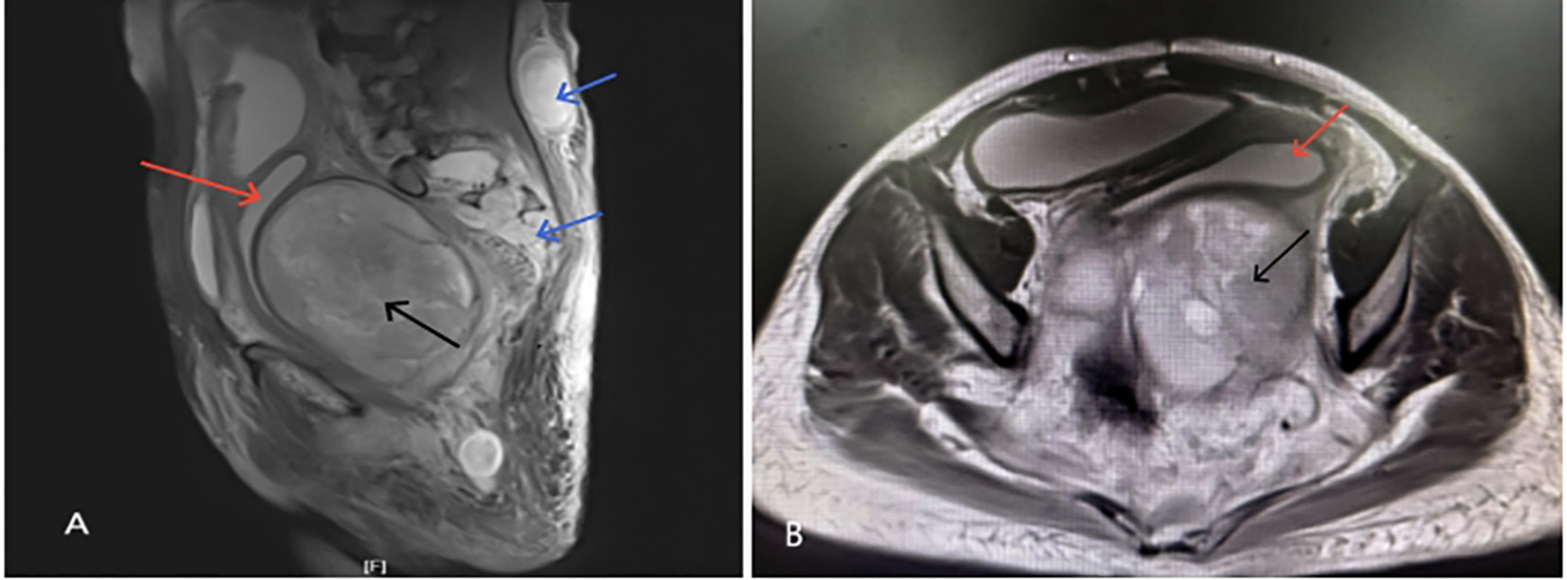
**(A, B)** Show irregular nodular mass (black arrow) ~112.5×96.7×119.2 mm below and behind the uterus(red arrow) with mixed T1 and long T2 signals; **(A)** shows nodular mass ~36.7×27.7×49.5 mm in the subcutaneous fat space at L4–5 level with unclear boundaries and nodular signals(blue arrow) in the sacrococcygeal canal and foramina.

- A large, irregular, mass-like lesion with mixed T1 long and T2 signals was observed posterior to the uterus, measuring approximately 112.5×96.7×119.2 mm, suggesting the possibility of a GN.
- A nodular mass, approximately 36.7×27.7×49.5 mm, was seen within the subcutaneous fat space of the lower back. Multiple nodular slightly long T2 signals were observed in the S2-S3 sacral canal and bilateral sacral foramina, with indistinct boundaries, uneven enhancement, and peripheral enhancement—possible neurofibromas?
- Fluid accumulation was also observed in the mid and lower segments of the uterine cavity and cervical canal (**Figure 2**).

### 2.4 MDT discussion

Given that the mass was located in the retroperitoneal pelvic region and involved the spinal canal, an MDT discussion was held, involving the orthopedics, urology, radiology, gastroenterology, oncology, and pathology departments. The MDT concluded the following:

- The enlarging mass could lead to severe bleeding, infection, and compression symptoms, indicating surgical intervention.
- The GN diagnosis had been confirmed by biopsy during an exploratory laparotomy at age 9. However, the mass had since grown into the sacral canal, sacral foramina, and subcutaneous space of the lower back, raising the possibility of malignant transformation.
- The surgery would be highly challenging with potential for significant intraoperative bleeding. Therefore, preoperative angiography was recommended to delineate the blood supply to the mass, followed by embolization to minimize intraoperative hemorrhage.
- Given that the mass was located between the vagina and rectum, a transvaginal approach for mass resection was recommended.

### 2.5 Surgical procedure (December 21, 2023)

On December 19, 2023, embolization was performed on the vessels supplying the mass. The procedure involved catheterization via the femoral artery and embolization of the posterior branch of the left internal iliac artery using absorbable gelatin sponge particles. On December 21, under general anesthesia, the mass in the vaginal rectal septum was removed via a transvaginal approach, followed by posterior vaginal wall repair and hysteroscopy. Intraoperatively, a large amount of purulent fluid was drained from the upper vagina, and repeated iodine solution irrigation was performed. A longitudinal incision approximately 8 cm long was made on the posterior vaginal wall near the hymenal ring. A large, well-defined, soft mass was visualized. The mass was bluntly dissected from the space between the vagina and rectum, ensuring no significant residual mass. Speculum examination showed significant dilation of the upper vagina, a flattened and congested cervix, and endometrial edema and congestion with inflammation on hysteroscopy.

### 2.6 Pathology

**Figure 3:**
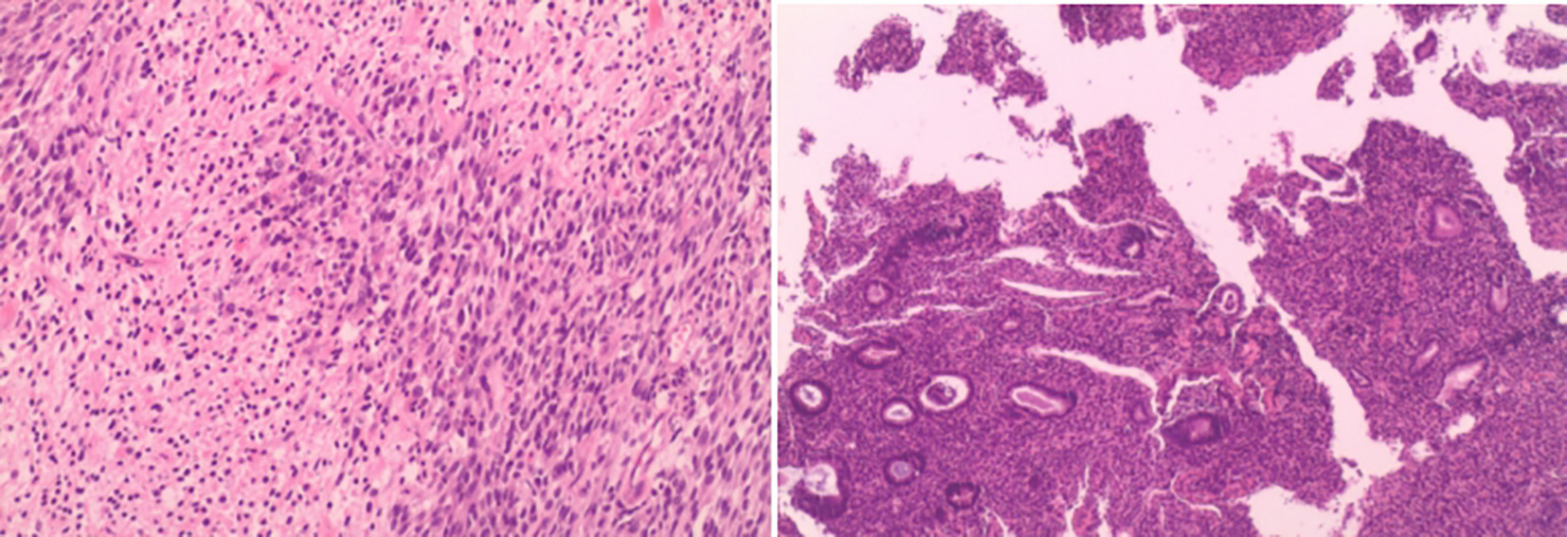
Cells are spindle-shaped with abundant fusiform cells, deeply stained nuclei with elongated, pointed, curved, or wavy shapes. Few mitoses observed. Dense cellular areas alternate with sparse areas.

Intraoperative rapid pathology suggested a recurrent neurogenic tumor with mild atypia and few mitotic figures. Postoperative pathology (**Figure 3**) indicated malignant transformation of neurofibromatosis (with malignant components as MPNST). Immunohistochemistry results were as follows: Tumor cells in paraffin block #17 included H3K27me3 (-, indicating loss), SOX10 (sporadic +), S-100 (sporadic +), CD10 (±), CD34 (–), and Ki-67 (~20% +). Tumor cells in paraffin block #11 included H3K27me3 (focal -, indicating possible loss), S-100 (+), and SOX10 (scattered +). Targeted immunohistochemistry showed that approximately 40% of tumor cells were pan-TRK positive in hotspot areas. In addition, chronic endometritis was noted.

## 3 Discussion

NF1 is an autosomal dominant hereditary disease, with half of cases inherited from affected family members, while the remainder result from *de novo* mutations in the NF1 gene located on chromosome 17q11.2. The NF1 gene is considered one of the genes with the highest spontaneous mutation rates (9). The incidence of NF1 in the general population is approximately 1 in 3,000, with approximately 8%–13% progressing to MPNST, which is the leading cause of mortality in patients with NF1 (9, 10). Studies have shown that patients with NF1 with deep neurofibromas are more prone to malignant transformation, whereas superficial neurofibromas rarely undergo such changes (11). The survival rate for NF1-associated MPNST is lower than sporadic MPNST (12). The NF1 gene is considered a tumor suppressor gene, and patients diagnosed with NF1 have an increased risk of developing gliomas, sarcomas, ovarian cancer, breast cancer, and melanoma, with a tendency for these conditions to occur at younger ages (1, 12, 13). NF1 typically manifests in childhood, with almost all patients showing symptoms before the age of 20, characterized by multiple café-au-lait spots and neurofibromas. Other common features include axillary and inguinal freckling, Lisch nodules, and optic nerve gliomas (3). In the case presented, the patient and her son exhibited typical café-au-lait spots, and genetic testing confirmed the diagnosis. According to distribution characteristics, neurofibromas present as dermal, spinal nerve root, nodular, and plexiform neurofibromas (pNFs), with dermal neurofibromas being the most common and having minimal risk of malignancy, while the latter two types are associated with an increased risk of malignancy (3, 14). Dermal neurofibromas are widely distributed, allowing for observation, with surgery considered when necessary. Nodular neurofibromas are discrete masses in subcutaneous or deep tissues that generally do not invade surrounding tissues but may cause pain or functional deficits like spinal nerve root neurofibromas (3). As seen in this case, multiple nodular neurofibromas were found in the subcutaneous fat, puborectal space, and sacral canal, compressing surrounding nerves and causing lumbosacral pain. PNFs grow along nerve plexuses and can involve extensive regions such as the head and neck, limbs, abdomen, pelvis, and peripheral nerves. Compared to the other types, pNF has the highest risk of malignant transformation, and large, extensive MPNSTs are often associated with widespread pNFs (14). NF1 can also evolve into atypical neurofibromatous neoplasms of uncertain biological potential (ANNUBP), which are considered the closest precursor lesions to MPNST (15). Surgical intervention is recommended for nodular neurofibromas and pNFs when clinical symptoms arise, malignancy is suspected, or the tumor size exceeds 6 cm. However, the large size of tumors, rich blood supply, and potential risk of damaging surrounding vital nerves increase surgical difficulty. Some scholars believe that the younger the patients, the higher the likelihood of recurrence, so they recommend delaying surgery until adolescence when the condition has stabilized, while others advocate for early surgery when the tumor is small to achieve complete excision and prevent malignant transformation (14). In summary, the presence of deep neurofibromas or pNFs warrants caution regarding potential malignancy. In this case, the pelvic mass previously identified as GN exhibited malignant transformation to MPNST on the background of neurofibromas, which indicated a complex tumor composition of both GN and deep neurofibromas.

GN originates from the neural ectoderm and is a rare, well-differentiated, and slowly growing benign peripheral nerve tumor composed of mature ganglion cells and Schwann cells. With an incidence of 0.0001%, it is most commonly observed in children with an average age of 7 years. Approximately 60%–80% of GN occurs in the posterior mediastinum or retroperitoneum, with the remaining cases being found in various locations, such as the adrenal glands and pelvis (16). These tumors are typically asymptomatic and often detected as large masses during routine physical exams, with an average diameter of 8 cm. They tend to grow along the direction of sympathetic nerve fibers and exhibit a characteristic “pseudopod” or “droplet” appearance on imaging due to their embedded growth pattern. The probability of malignancy is very low, typically progressing to neuroblastoma, although rare cases of malignant transformation to MPNST have been reported (5). CT and MRI provide diagnostic value in part, however, fine-needle aspiration is necessary for definitive diagnosis of GN. The preferred treatment is surgical resection, aiming for complete removal while preserving normal nerve function. In this case, the patient was diagnosed with a pelvic GN measuring over 7 cm that was not surgically removed initially at a young age. Later, the patient was found to have NF1, and differentiating between GN and neurofibromatosis on imaging was challenging. The tumor gradually enlarged, causing pain and significant compressive symptoms, raising important considerations about the timing of surgery, surgical approach, and early detection of malignant transformation. Secondary MPNSTs associated with NF1 and those of unknown etiology account for nearly half of all cases, with a small proportion linked to radiation exposure. MPNSTs most commonly arise in the peripheral nerves of the limbs and pelvis and often metastasize to the lungs (17, 18). Rapid changes in tumor size or the onset of pain may indicate malignant transformation (19). The 18F-fluorodeoxyglucose (18F-FDG) PET/CT has shown sensitivity and specificity in detecting MPNST. The intensity of FDG uptake correlates with tumor grading and can be used not only to monitor disease progression in NF1 but also for postoperative surveillance (19, 20).

In this case, the primary symptoms involved significant vaginal bleeding and chronic compression symptoms. Despite treatments with Uroxatral and Diane-35, the patient continued to experience intermittent heavy bleeding. Vaginal bleeding was suspected to be non-uterine in origin, and the large tumor caused vaginal obstruction and difficulty in expelling vaginal secretions. As a result, a long-standing infection and accumulation of pus occurred in the upper vagina, leading to its dilation. Chronic inflammatory responses and tumor compression caused vascular proliferation in the posterior vaginal wall, with recurrent ruptures of these proliferated vessels leading to repeated heavy bleeding. The enlarging tumor compressed surrounding nerves, causing lumbosacral pain, left lower limb pain, and left-sided vulvar edema due to obstructed venous return from the left pelvic area.

Based on the previous biopsy and MRI findings, the rectovaginal septum tumor was considered a large GN. Prior to surgery, in addition to symptomatic treatments, such as blood transfusion for anemia, and piperacillin for infection, performing preoperative embolization of the dominant blood vessels supplying the tumor was crucial because of the rich vascularity and high risk of bleeding. The tumor, located in the rectovaginal septum, could be excised either via an abdominal or vaginal approach. Although abdominal surgery provides better surgical visibility, it causes.significant drawbacks including greater surgical trauma, blood loss, and prolonged recovery. In contrast, the transvaginal approach offers minimally invasive access, expedited recovery, and direct proximity to the lesion. Thus, We chose transvaginal approach. We were fully aware that the restricted surgical field inherent to this approach could potentially lead to residual tumor, tumor seeding, or injury to adjacent organs. Owing to the tumor size and location, there were risks of incomplete excision, hemorrhagic shock, nerve and adjacent organs damage, all of which could impact the patient’s daily life. Additionally, because GN is a benign lesion with controllable symptoms, major hospitals advise against surgery and opt for symptomatic treatment. Although the tumor was considered a GN preoperatively, surgical treatment was unavoidable due to the presentation of severe, refractory symptoms. However, postoperative pathology revealed an MPNST arising from neurofibromas, indicating that the tumor comprised not only GN but also neurofibromas. The prolonged conservative treatment may have led to the malignant transformation of neurofibromas. Neurofibromas belong to peripheral nerve sheath tumors, whereas ganglioneuromas are benign lesions located at the ganglion, and both are classified as peripheral nerve disorders. Definitive diagnosis of MPNST relies on histopathology. Immunohistochemical findings typically demonstrate: H3K27me3 (–), S-100/SOX10 (sporadic +). Loss of H3K27me3 is a diagnostic marker for MPNST and an indicator for an inferior survival, which can be observed in 60% of NF1-associated MPNSTs and 90% of sporadic, radiation-induced MPNSTs (21, 22). S-100/SOX10 are Schwann cell markers, and their expression is diffuse in NF1 but partial loss in MPNST (23). MPNST carries a high-grade malignancy with 50% 5-year mortality. The only curative approach is R0 surgical resection (negative margins), yet postoperative recurrence reaches 30-70% (24). Adjuvant chemotherapy and radiotherapy remain controversial. Currently, adjuvant chemotherapy is often used for inoperable or metastatic MPNSTs, and doxorubicin or epirubicin combined with ifosfamide has shown relative efficacy (25). Although radiotherapy is a known risk factor for inducing MPNSTs, it can improve local recurrence rates to some extent. Thus, after weighing the risks and benefits, radiotherapy may be considered (20). Targeted therapies show promise, such as MEK/mTOR inhibitors.Inhibiting PRC2 loss is also a key target, such as HDAC/DNMT1inhibitors (15). MEK inhibitors selumetinib also could be used to shrink unresectale neurofibromas (23). In this case, genetic testing (e.g.,NTRK) could guide targeted therapy to enhance 5-year survival. However, the patient did not undergo adjuvant therapy due to financial barriers to control the remaining deep neurofibromas and MPNSTs. Although postoperative tumor markers declined (HE4: 51 pmol/L; CA724: 7.41 U/mL) compared to preoperative levels, overall survival was less than 12 months.

## 4 Conclusion

This patient had both GN and NF1, two rare benign conditions. The risk of malignant transformation of GN is exceedingly rare; however, the likelihood of malignant transformation in NF1 with deep neurofibromas increases. MRI has limited specificity in differentiating GN from neurofibromas and detecting NF1 progressing to MPNST. Given the poor prognosis of MPNST secondary to NF1, timely surgical intervention of NF1 to prevent malignancy and early detection of malignancy are crucial. FDG-PET/CT has some value in detecting MPNST. Once MPNST is diagnosed, surgical treatment is the first choice, followed by adjuvant radiotherapy and chemotherapy. Research on targeted therapies is currently very active; however, long-term studies and clinical data are needed to confirm their efficacy.

## Data Availability

The raw data supporting the conclusions of this article will be made available by the authors, without undue reservation.
